# Evaluation of the immunization efficacy and adverse reactions of hepatitis B vaccination in children with thalassemia minor

**DOI:** 10.1186/s12889-024-18779-1

**Published:** 2024-09-27

**Authors:** Xue Han, Xi Zhang, Liling Zhong, Ying Liu, Lifen Gong, Jikai Zhang, Hai Wang, Qingsong Chen

**Affiliations:** 1 School of Public Health, Guangdong Pharmaceutical University, Guangzhou, 510310 China; 2 Heyuan Center for Disease Control and Prevention, Heyuan, China; 3 Guangdong Provincial Institute of Biological Products and Materia Medica, Guangzhou, China

**Keywords:** Adverse reaction, Hepatitis B vaccination, Immunization, Thalassemia

## Abstract

**Objective:**

To assess the immunization efficacy and incidence of adverse reactions after hepatitis B vaccination in children with thalassemia based on data from real-world studies.

**Methods:**

A total of 625 children were recruited into this cross-sectional study. Subgroup analyses of different thalassemia types were performed using binary logistic regression, the factors affecting HBsAb levels were identified using multiple linear regression, and the dose-response relationship between the duration of immunization and seroconversion was explored using the restricted cubic spline (RCS) model to further assess the protective duration of the hepatitis B vaccine.

**Results:**

HBsAb positivity in enrolled children was 87.3% in the thalassemia group and 81.4% in the control group. Multifactorial analysis revealed that the duration of immunization, age at completion of vaccination, and whether the first dose was delayed were significant factors influencing HBsAb levels in children (*P* < 0.05). The threshold for HBsAb positivity may be reached when the immunization duration reaches approximately 30 months. A subgroup analysis revealed that the HBsAb positivity rate was lower in children with β-thalassemia minor compared to those with α-thalassemia minor (*P* = 0.001, 95% CI: 0.097 ∼ 0.536). Adverse reactions after hepatitis B vaccination were dominated by general reactions, with a statistically significant difference in injection-site redness and swelling between the thalassemia and control groups (*P* < 0.05).

**Conclusions:**

The immunization response to the hepatitis B vaccine in children with thalassemia minor was comparable to healthy children, with no abnormal adverse effects seen.

**Supplementary Information:**

The online version contains supplementary material available at 10.1186/s12889-024-18779-1.

## Introduction

Thalassemia is a monogenic genetic disorder caused by genetic deletions or mutations and is prevalent worldwide [1]. China is one of the countries with a high prevalence of thalassemia, particularly in the regions south of the Yangtze River; the thalassemia gene carrier rate varies greatly among the different regions, ranging from 3.3 to 24.07%, with the highest rates in the Guangxi and Guangdong provinces [2]. The clinical manifestations of thalassemia vary widely among patients, ranging from no obvious clinical manifestations to mild microcytic hypochromic anemia in mild cases to regular blood transfusion therapy or even death in severe cases.

Based on current medical technology, hepatitis B is incurable; therefore, preventing hepatitis B virus (HBV) infection is especially vital. HBV vaccination is the most critical measure to prevent and control the prevalence of hepatitis B in China [3]. Although the hepatitis B vaccine for the prevention of HBV infection has been recommended for patients with thalassemia, these individuals remain at an increased risk of HBV infection, since their impaired immune function may result in decreased immunity even after receiving hepatitis B vaccination [4–8].

Hepatitis B vaccination programs have been in general use since 1982 and have shown protection rates of over 90% [9]. Studies have shown that in approximately 40% of vaccinated cases, individuals no longer test positive for hepatitis B surface antibody (HBsAb) seven years after vaccination [10]. Furthermore, this response rate may be significantly lower in patients with chronic diseases including thalassemia [11].

The Advisory Committee on Immunization Practices (ACIP) recommends routine doses of the hepatitis B vaccine for the general population; furthermore, they strongly recommend a booster dose for thalassemia patients with low antibody levels (< 100 IU/mL) [12]. Although hepatitis B vaccination can effectively reduce the rate of HBV infection in thalassemia patients, controversy remains among scholars regarding the immunization efficacy of hepatitis B vaccination in children with thalassemia; therefore, further verification is needed to determine whether the protective effect of hepatitis B vaccination is reduced in children with thalassemia relative to healthy children.

There are few studies on hepatitis B vaccination in children with thalassemia, most of which have small sample sizes. Therefore, in this study, we investigated the immune effects and occurrence of adverse reactions after vaccination in children with thalassemia, analyzed the influencing factors related to the level of HBsAb, and further predicted the immune persistence of completing three doses of the hepatitis B vaccine.

## Methods

### Study subjects

In this cross-sectional survey based on real-world data, hepatitis B vaccination information was collected from questionnaires that were administered to the parents of children aged 1 to 4 years between March 2022 and January 2023 in Heyuan City, Guangdong Province, China. The data mainly included basic demographic information, the occurrence of adverse reactions after vaccination, hepatitis B vaccination-related information, and the duration of immunization (defined as the interval between the date of blood collection and the date of the third dose of basic immunization with the hepatitis B vaccine).

Children enrolled in this study were screened among those who were registered and completed the free prenatal thalassemia screening and diagnosis program at the Maternal and Child Health Hospital in Heyuan during the period of 2018–2022. All enrolled children were screened and diagnosed for thalassemia before birth. In addition, the Guangdong Provincial Vaccine Circulation and Vaccination Management Information System was used to verify the three-dose of hepatitis B vaccine for the children in this study. Information verified included the type of hepatitis B vaccination, the dose, the time of vaccination, and the site of vaccination.

All children enrolled in this study had completed a three-dose recombinant hepatitis B vaccination schedule (one dose on the day of birth, one dose at 1 month of age, and one dose at 6 months of age). Children infected with HBV at birth, those suffering from immunodeficiency diseases, those who had not completed the three-dose hepatitis B vaccine, and those whose mothers had been infected with hepatitis B were excluded. All participants’guardians provided written informed consent. The study protocol complied with the Declaration of Helsinki 1964 and its later amendments.

After the children were enrolled, venous blood was collected in a 3-mL tube and centrifuged, and the separated serum was stored in a -80 °C cryogenic refrigerator. The serum HBsAb level was quantified using a chemiluminescent immunoassay. A serum HBsAb concentration ≥ 10 mIU/mL was considered antibody positive and effectively able to prevent HBV infection, while a serum HBsAb concentration < 10 mIU/mL was considered antibody negative [13].

### Statistical analysis

Count data were statistically described using frequency and constitutive ratio (rate). Count data and rate comparisons were made using the chi-square test and trend chi-square test; measurement data comparisons were made using the two independent samples *t*-test. Multiple linear regression was used for multifactorial analysis and binary logistic regression analysis was used for subgroup analysis. Restricted cubic spline (RCS) was plotted using R Version 4.1.0 software to analyze the dose-response relationship between the key variables and HBsAb levels. Statistical analysis was performed using SPSS Version 25.0 software.

## Results

### Demographic and clinical characteristics

A total of 625 children aged 1 to 4 years were recruited in this study, including 307 children with thalassemia and 318 healthy children. All the enrolled children were given three 10 µg doses of recombinant hepatitis B vaccine intramuscularly after birth according to the immunization program schedule.

The enrolled children with thalassemia were divided into five groups based on the type of thalassemia: α-thalassemia and β-thalassemia. These groups were further categorized based on sub-type. There were three types of α-thalassemia (silent carrier-α, minor-α, and intermedia-α) and one type of β-thalassemia (minor-β), with the smallest group being α-thalassemia intermedia with eight children. There was also a group consisting of four children with a combination of α-thalassemia minor and β-thalassemia minor. Among the enrolled children, 527 were HBsAb positive—268 in the thalassemia group and 259 in the control group—and there was a statistically significant difference between the two groups in terms of HBsAb positivity (*P* = 0.044). See Table 1 for details.

**Table 1:** Demographic characteristics of participants

Characteristic		Total (*N* = 625)	Thalassemia group (*N* = 307)					Control group (*N* = 318)	*P*
			silent carrier-α (*N* = 56)	minor-α (*N* = 166)	Intermedia (*N* = 8)	minor-β (*N* = 73)	minor-α combined with minor-β(*N* = 4)		
Age, years									**0.042**
	1∼	133 (21.3)	16 (28.6)	39 (23.5)	3(37.5)	11 (15.1)	1(25.0)	63 (19.8)	
	2∼	167 (26.7)	16 (28.6)	49 (29.5)	5(62.5)	16 (21.9)	3(75.0)	78 (24.5)	
	3∼	163 (26.1)	12 (21.4)	51 (30.7)	0(0.0)	21 (28.8)	0(0.0)	79 (24.8)	
	4∼	162 (25.9)	12 (21.4)	27 (16.3)	0(0.0)	25 (34.2)	0(0.0)	98 (30.8)	
Sex									0.296
	Boy	341 (54.6)	34 (60.7)	85 (51.2)	4(50.0)	48 (65.8)	3(75.0)	167 (52.5)	
	Girl	284 (45.4)	22 (39.3)	81 (48.8)	4(50.0)	25 (34.2)	1(25.0)	151 (47.5)	
Premature									0.264
	No	587 (93.9)	53 (94.6)	156 (94.0)	6(75.0)	66 (90.4)	4(100.0)	302 (95.0)	
	Yes	38 (6.1)	3 (5.4)	10 (6.0)	2(25.0)	7 (9.6)	0(0.0)	16 (5.0)	
Delayed first vaccination									0.569
	Yes	51 (8.2)	2 (3.6)	21 (12.7)	1(12.5)	3 (4.1)	0(0.0)	24 (7.5)	
	No	574 (91.8)	54 (96.4)	145 (87.3)	7(87.5)	70 (95.9)	4(100.0)	294 (92.5)	
Age at completion of vaccination									0.756
	< 7 months old	373 (59.7)	31 (55.4)	100 (60.2)	5(62.5)	48 (65.8)	2(50.0)	187 (58.8)	
	≥ 7 months old	252 (40.3)	25 (44.6)	66 (39.8)	3(37.5)	25 (34.2)	2(50.0)	131 (41.2)	
HBsAb									**0.044**
	-	98 (15.7)	7 (12.5)	11 (6.6)	1(12.5)	20 (27.4)	0(0.0)	59 (18.6)	
	+	527 (84.3)	49 (87.5)	155 (93.4)	7(87.5)	53 (72.6)	4(100.0)	259 (81.4)	

### Distribution of HBsAb positivity by duration of immunization

When the duration of immunization was < 1 year, the HBsAb positivity rate of the control group was slightly higher than that of the thalassemia group, 97.3% and 96.7%, respectively, but the differences were not statistically significant (*P* > 0.05). In all other groups with durations of immunization ≥ 1 year, the HBsAb positivity rate of the thalassemia group was higher than that of the control group, but none of the differences were statistically significant (*P* > 0.05). See Table 2 for details.

**Table 2:** HBsAb positivity in children by duration of immunization

Duration of immunization		Total (*N* = 625)	HBsAb		χ^2^	*P*
			+ (*N* = 527)	- (*N* = 98)		
< 1 year					0.023	0.880
	Thalassemia group	30	29 (96.7)	1 (3.3)		
	Control group	37	36 (97.3)	1 (2.7)		
∼ 1 year					0.001	0.985
	Thalassemia group	100	95 (95.0)	5 (5.0)		
	Control group	79	75 (94.9)	4 (5.1)		
∼ 2 years					0.359	0.549
	Thalassemia group	86	80 (93.0)	6 (7.0)		
	Control group	73	66 (90.4)	7 (9.6)		
∼ 3 years					0.986	0.321
	Thalassemia group	67	48 (71.6)	19 (28.4)		
	Control group	95	61 (64.2)	34 (35.8)		
∼ 4 years					0.146	0.702
	Thalassemia group	24	16 (66.7)	8 (33.3)		
	Control group	34	21 (61.8)	13 (38.2)		

### Analysis of immune response to hepatitis B vaccine in children with different thalassemia types

The results showed a statistically significant difference in immune response between the different thalassemia types when the duration of immunization was between 3 and 4 years (χ^2^ = 15.881, *P* = 0.006). Further comparisons revealed that the difference in immune response between α-thalassemia minor and β-thalassemia minor was statistically significant (χ^2^ = 13.969, *P* = 0.002). See eTable 1 in Supplement 1.

### Analysis of factors influencing the level of HBsAb

The univariate analysis results revealed that the duration of immunization, delay in the first dose, and age at completion of vaccination affected the level of HBsAb (*P* < 0.05), while the presence of thalassemia was not found to affect the level of HBsAb (*P* > 0.05). See Table 3 for details.

**Table 3:** Univariate analysis of the distribution of HBsAb levels in children

Characteristic		Total (*N* = 625)	GMC (mIU/mL)	F	*P*
Sex				0.367	0.565
	Boy	341 (54.6)	81.70		
	Girl	284 (45.4)	82.08		
Thalassemia				0.545	0.461
	Yes	307 (49.1)	92.21		
	No	318 (50.9)	72.99		
Duration of immunization				73.753	**< 0.001**
	< 1year	67 (10.7)	496.02		
	∼ 1 year	179 (28.6)	225.89		
	∼ 2 years	159 (25.4)	94.38		
	∼ 3 years	162 (25.9)	20.62		
	∼ 4 years	60 (9.6)	14.20		
Delayed first vaccination				14.889	**< 0.001**
	Yes	51 (8.2)	174.26		
	No	574 (91.8)	76.56		
Age at completion of vaccination				1.335	**0.013**
	< 7 months old	373 (59.7)	78.11		
	≥ 7 months old	252 (40.3)	87.77		

The multiple linear regression analysis results revealed that age at the time of completion of three doses of hepatitis B vaccination in children was an independent influence on HBsAb levels (*P* = 0.012). The duration of immunization was also a significant influence on HBsAb levels (*P* < 0.001), with antibody levels decreasing as the duration of immunization increased. In addition, we also found that the antibody level was higher in children whose first vaccination was delayed (*P* = 0.030), whereas sex and the presence of thalassemia did not affect the level of HBsAb (*P* > 0.05). See Table 4 for details.

**Table 4:** Multiple linear regression analysis of HBsAb levels in children

Characteristic	Unstandardized Coefficients		Standardized Coefficient	t	*P*
	B	SE	Beta		
Duration of immunization	-165.844	11.448	-0.524	-14.487	**< 0.001**
Age at completion of vaccination	14.781	5.862	0.084	2.522	**0.012**
Delayed first vaccination	92.870	42.697	0.072	2.175	**0.030**
Thalassemia	-15.655	23.314	-0.022	-0.671	0.502
Sex	16.172	23.267	0.023	0.695	0.487

To further explore whether there were differences in seroconversion in children with different thalassemia types, subgroup analyses were done. Considering that different severities of thalassemia would affect the accuracy of the results, only children with α-thalassemia minor and β-thalassemia minor were included. After adjusting for confounding factors, it was found that the antibody positivity rate was lower in those with β-thalassemia minor compared to α-thalassemia minor (*P* = 0.001, 95% confidence interval (CI): 0.097∼0.536). See eTable 2 in Supplement 1.

**Fig. 1:**
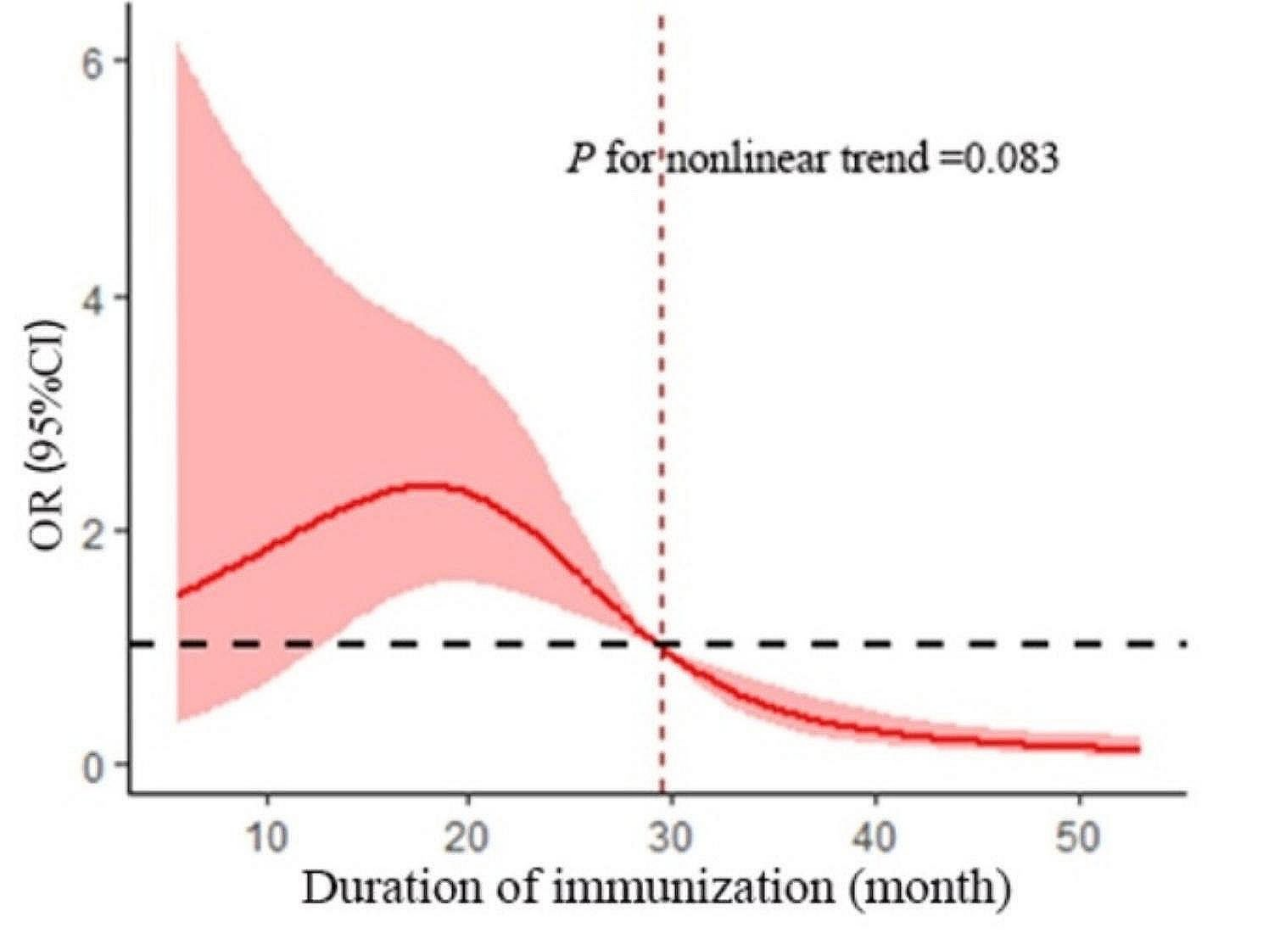
The RCS model of duration of immunization and seroconversion

To further explore immune persistence after childhood vaccination, a four-node restricted cubic spline (RCS) model based on binary logistic regression analysis was used to fit the dose-response relationship between the duration of immunization and seroconversion. Solid lines represent odds ratios (ORs) and shaded areas show the 95% CIs. The results showed a linear dose-response relationship (*P*_non−linear_ > 0.05, *P*_total_ < 0.05) between the duration of immunization and seroconversion. When the duration of immunization reached approximately 30 months, the antibody was no longer protective, and the antibody-positive threshold was reached. See Fig. 1.

### Occurrence of adverse reactions after hepatitis B vaccination in children

According to the degree of damage to the organism, vaccine adverse reactions are categorized into general reactions and abnormal reactions. In this study, all the adverse reactions that occurred after hepatitis B vaccination in all the enrolled children were general reactions, and there were no abnormal reactions.

Parents of 24 of the children reported adverse reactions after their children received the hepatitis B vaccine, which included four main conditions: redness and swelling, induration, pain at the vaccination site, and fever. The chi-square test results showed that there was a statistical difference between the thalassemia group and the control group in terms of redness and swelling at the vaccination site (*P* < 0.05). The vast majority of adverse reactions in children after hepatitis B vaccination resolved on their own in approximately three days with physical cooling or no treatment. See Table 5 for details.

**Table 5:** Adverse reactions after hepatitis B vaccination in children

Reported reaction	First dose		*P*	Second dose		*P*	Third dose		*P*
	Thalassemia group (*N* = 307)	Control group (*N* = 318)		Thalassemia group (*N* = 307)	Control group (*N* = 318)		Thalassemia group (*N* = 307)	Control group (*N* = 318)	
Injection-site redness and swelling	3 (1.0)	13 (4.1)	**0.014**	1 (0.3)	9 (2.8)	**0.021**	0 (0.0)	8 (2.5)	**0.008**
Injection-site induration	2 (0.7)	7 (2.2)	0.177	0 (0.0)	2 (0.6)	0.499	0 (0.0)	2 (0.6)	0.499
Fever	0 (0.0)	0 (0.0)	-	1 (0.3)	1 (0.3)	1.000	0 (0.0)	1 (0.3)	1.000
Injection-site pain	0 (0.0)	3 (0.9)	1.000	0 (0.0)	3 (0.9)	0.249	0 (0.0)	2 (0.6)	0.499

We then used binary logistic regression to further analyze the independent factors affecting the occurrence of adverse reactions after hepatitis B vaccination. The results showed that having thalassemia was an independent factor affecting children after both the first and second doses of hepatitis B vaccination (*P* < 0.05). See eTable 3 in Supplement 1.

## Discussion

Between 2000 and 2019, administration of three doses of the hepatitis B vaccine in newborns has increased from 29 to 85% globally [14]. To date, although the rate in China of administering the full course of the hepatitis B vaccine has been maintained at more than 95% [15], China still has the highest rate of HBV infection in the world [16], and is also a major country for hepatitis B vaccine use. Effective implementation of hepatitis B vaccination programs has resulted in significant reductions in HBV carriage and hepatitis B-related morbidity and mortality, and vaccination remains a cost-effective population-wide intervention to achieve elimination of the disease.

However, it is inconclusive whether the effect of hepatitis B vaccination in children with certain special disease conditions is different from that of healthy children. Therefore, it is important to explore the effectiveness of hepatitis B vaccination in children with thalassemia in view of the high utilization of the hepatitis B vaccine in China.

Since its introduction, the hepatitis B vaccine has been widely recognized for its good immunogenicity and safety. However, many factors affect the immunogenicity of the hepatitis B vaccine; in this study, the duration of immunization, age at the completion of vaccination, and whether the first dose was delayed were found to be independent influences affecting the level of HBsAb in children.

Although it is well known that the protective effect of hepatitis B vaccination wanes with increasing duration of immunization, the duration of the protective effect is not fully understood. Among children who complete the basic immunization series and develop protective antibodies, 15–50% have low or even undetectable concentrations of HBsAb 5–15 years after vaccination [13]. However, some studies have suggested that even if the serum antibody level becomes negative, memory immunity remains in the body with a protective effect that lasts for up to 10 years [17]. Re-immunization of individuals who turn antibody-negative is not currently recommended by the WHO [18], possibly due to insufficient data at this time to support this recommendation, or due to economic considerations. However, more studies have shown that children who are antibody-negative after completing the three-dose basic immunization series as newborns are likely to have lost their humoral memory immunity and be at risk for HBV infection [19]. The present study showed that children reached the threshold of antibody positivity approximately 30 months after completing the whole course of immunization and may no longer be protected. For people at high risk of HBV infection, such as people living with family members who are hepatitis B surface antigen (HBsAg)-positive, it is recommended that they be tested regularly for HBsAb and receive booster dose according to their antibody levels.

According to our immunization program protocol, the third dose of hepatitis B vaccine should be completed at six months of age. However, there may be cases when the third vaccination dose is delayed when additional factors are considered, such as the child’s health condition and the influence of the general social environment. According to the results of the present study, this delayed vaccination may not negatively affect the immunization outcome of children. A study as early as 1995 also showed that delaying the third dose of vaccine for two years in newborns did not affect the level of immune response [20]. Delaying the third dose of the hepatitis B vaccine for months or even years not only does not affect the immune response, but also improves the antibody positivity rate relative to the routine vaccination regimen [21], which is consistent with our study that showed that children who received the third dose of the vaccine at a later date demonstrated higher levels of HBsAb. Additionally, in subgroup analyses, children who completed the vaccination series after seven months of age had a significantly higher rate of HBsAb positivity than those below that age.

Since perinatal and early postnatal transmission is the leading cause of chronic hepatitis infection, the WHO recommends that all newborns be vaccinated with the hepatitis B vaccine within 24 h after birth [22]. However, in neonates born to HBsAg-negative mothers, delaying the first vaccination may improve immunization.

In this study, it was found that children with a delayed first vaccination had higher antibody levels, which was an important factor influencing the antibody levels in children after completing the full course of vaccination. A study by Agladioglu et al. [23] found that children who received the first dose of the hepatitis B vaccine with a two-month delay had an immune response rate of 94.9%, compared to 57.7% for those who received the vaccine immediately after birth. Instead of immunizing newborns at birth, some countries are now choosing to screen pregnant women for HBsAg and only immunize infants at birth who are born to HBsAg-positive mothers [22]. In some countries, such as Turkey, the first hepatitis B vaccination for infants born to HBsAg-negative mothers can be delayed until a few months after birth, the same time as the first vaccination for DTaP-IPV/Hib and pneumococcal conjugate vaccines [23].

In 1992, 60 patients with thalassemia major and 60 healthy controls who had completed three doses of hepatitis B vaccination after birth were included in an Italian study [24]. After five years of follow-up, the antibody concentrations were higher in the healthy control group than in the thalassemia group; however, the difference was not statistically significant between the two groups, probably due to the small sample size of the study. In 2021, a report from India on children with thalassemia major who had been vaccinated against hepatitis B for more than five years had a significantly higher seroprotection rate than healthy children (*P* < 0.001) [25]. This phenomenon has been interpreted to be a result of prolonged transfusion therapy, whereby increased antigenic stimulation occurs to generate stronger immune responses as well as increased antibody transport through the donor’s blood resulting from passive transportation. In addition to this, other vaccine-related studies on thalassemia patients vaccinated with Haemophilus influenzae type b conjugate, 13-valent pneumococcal conjugate, influenza C cerebral conjugate, and tetanus vaccines have found similarly good immunogenicity and safety after vaccination [26–29].

In the present study, it was found that four years after completing all three doses of the hepatitis B vaccination series, the thalassemia group had a slightly higher rate of HBsAb positivity than the control group, 66.7% and 61.8%, respectively, but the difference between the two groups was not statistically significant. The multifactorial analysis results showed that having thalassemia did not affect the level of HBsAb, which may be related to the type of thalassemia we included. Due to the widespread availability of prenatal genetic testing programs for mothers, mothers with fetuses with thalassemia major or intermedia often choose to terminate their pregnancies, leading to an extremely low birth rate of children with thalassemia major. Therefore, the limitation of this study is that most of the children included had only silent carrier or minor types of thalassemia, which may present with only mild anemia that does not severely affect autoimmune function. The age range of enrolled children needs to be further expanded in future studies to include more children with thalassemia major to evaluate the safety and immune efficacy of hepatitis B vaccination.

In this study, we found that the antibody positivity rate was lower in those with β-thalassemia minor compared to α-thalassemia minor, which may be due to differences in gene mutations. Studies have shown that α-thalassemia minor and β-thalassemia minor are caused by two gene deletions on chromosome 16 and one gene defect on chromosome 11, respectively, and neither has significant clinical symptoms. But two gene defects on chromosome 11 can cause intermediate β-thalassemia and show clinical symptoms of varying severity [30]. To our knowledge, this may be the first study to report this phenomenon, and future prospective cohort studies with larger sample size are needed for further validation.

Numerous clinical trials and extensive practical applications have proven that the hepatitis B vaccine is very safe. Abnormal adverse events following hepatitis B vaccination are uncommon and usually minor and transient. Most of the adverse events reported included mild symptoms at the injection site, temporary discomfort, fatigue, headache, nausea, rash, dizziness, arthralgia, myalgia, and somnolence, which resolved within two to three days [31]. In this study, the incidence of adverse reactions after vaccination in the enrolled children was 3.8%. In both groups, the adverse reactions were mainly fever and injection-site redness and swelling, pain, and induration, which resolved independently in approximately three days with physical cooling or no treatment. Studies on hepatitis B vaccination in children with thalassemia also found no abnormal adverse reactions [32], and the hepatitis B vaccine is equally desirable for thalassemia patients.

The Centers for Disease Control and Prevention (CDC) suggests that children and adults with thalassemia should receive all recommended vaccines. Meanwhile, *Consensus of experts on diagnosis and treatment of non-transfusion dependent thalassemia in Children (2018 edition)* also suggests that standardizing vaccination, preventing, and promptly treating infections are of great importance in reducing complications and maintaining normal growth and development in children [33]. Therefore, timely routine vaccination of children with thalassemia is necessary and feasible.

## Conclusions

Our study found that children with thalassemia minor showed equally good immunization with the hepatitis B vaccine compared to healthy children. There was a significant difference between the two groups in terms of redness and swelling at the vaccination site, and no other abnormal adverse reactions were seen.

## Electronic supplementary material

Below is the link to the electronic supplementary material.

Supplementary Material 1

Supplementary Material 2

## Data Availability

The data that support the findings of this study are avvailable from the corresponding author upon reasonable request.

## Abbreviations

ACIP: Advisory Committee on Immunization Practices
CI: Confidence interval; CDC: Centers for Disease Control and Prevention
HBV: Hepatitis B virus
HBsAb: Hepatitis B surface antibody
HBsAg: Hepatitis B surface antigen
OR: Odd ratio
RCS: Restricted cubic spline
