# Supplementary material for: Evaluation of the immunization efficacy and adverse reactions of hepatitis B vaccination in children with thalassemia minor
Source: BMC Public Health. 2024 Sep 27;24:2641. doi: 10.1186/s12889-024-18779-1 (PMC11438186; doi:10.1186/s12889-024-18779-1)
Supplement: Supplementary file 1 — Supplementary Material 1 [file 12889_2024_18779_MOESM1_ESM.doc]

**Supplemental Online Content**

**eTable 1**. Hepatitis B vaccine immune response in children with different types of thalassemia

**eTable 2**. Subgroup analysis of seroconversion in children with different thalassemia types

**eTable 3**. Binary logistic regression analysis of the occurrence of adverse reactions after hepatitis B vaccination in children

**eTable 1. Hepatitis B vaccine immune response in children with different types of thalassemia**

| Duration of immunization | Thalassemia types | HBsAb (mIU/mL) | | | | χ^2^ | *P* |
| --- | --- | --- | --- | --- | --- | --- | --- |
|  |  | < 10 | 10-100 | 100-1000 | > 1000 |  |  |
| < 1 year |  |  |  |  |  | 12.431 | 0.124 |
|  | silent carrier-a | 0 | 0 | 1 | 5 |  |  |
|  | minor-a | 0 | 0 | 7 | 11 |  |  |
|  | intermedia-a | 0 | 0 | 1 | 2 |  |  |
|  | minor-β | 1 | 0 | 1 | 0 |  |  |
|  | minor-a combined with minor-β | 0 | 0 | 1 | 0 |  |  |
| 1～2 years |  |  |  |  |  |  |  |
|  | silent carrier-a | 0 | 1 | 16 | 5 | 11.532 | 0.395 |
|  | minor-a | 2 | 10 | 29 | 10 |  |  |
|  | intermedia-a | 1 | 1 | 2 | 0 |  |  |
|  | minor-β | 2 | 4 | 12 | 2 |  |  |
|  | minor-a combined with minor-β | 0 | 1 | 2 | 0 |  |  |
| 2～3 years |  |  |  |  |  |  |  |
|  | silent carrier-a | 1 | 3 | 8 | 1 | 6.923 | 0.792 |
|  | minor-a | 4 | 20 | 25 | 2 |  |  |
|  | intermedia-a | 0 | 1 | 0 | 0 |  |  |
|  | minor-β | 1 | 9 | 9 | 2 |  |  |
|  | minor-a combined with minor-β | - | - | - | - |  |  |
| 3～4 years |  |  |  |  |  |  |  |
|  | silent carrier-a | 5 | 4 | 2 | 0 | 15.881 | **0.006** |
|  | minor-a | 4 | 20 | 12 | 1 |  |  |
|  | intermedia-a | - | - | - | - |  |  |
|  | minor-β | 10 | 5 | 2 | 2 |  |  |
|  | minor-a combined with minor-β | - | - | - | - |  |  |
| 4～5 years |  |  |  |  |  |  |  |
|  | silent carrier-a | 1 | 2 | 1 | 0 | 9.555 | 0.066 |
|  | minor-a | 1 | 8 | 0 | 0 |  |  |
|  | intermedia-a | - | - | - | - |  |  |
|  | minor-β | 6 | 3 | 1 | 1 |  |  |
|  | minor-a combined with minor-β | - | - | - | - |  |  |

**eTable 2. Subgroup analysis of seroconversion in children with different thalassemia types**

| Characteristic |  | OR | 95% CI | *P* |
| --- | --- | --- | --- | --- |
| Thalassemia types |  |  |  | **0.001** |
|  | minor-α | 1.00 |  |  |
|  | minor-β | 0.228 | 0.097～0.536 |  |
| Age at completion of vaccination |  |  |  | 0.076 |
|  | < 7 months old | 1.00 |  |  |
|  | ≥ 7 months old | 2.474 | 0.911～6.715 |  |
| Delayed first vaccination |  |  |  |  |
|  | No | 1.00 |  | 0.606 |
|  | Yes | 1.782 | 0.199～15.975 |  |
| Duration of immunization |  | 0.398 | 0.253～0.626 | < 0.001 |

**eTable 3. Binary logistic regression analysis of the occurrence of adverse reactions after hepatitis B vaccination in children**

| Dose |  | Characteristic | β | Wald | OR | 95% CI | *P* |
| --- | --- | --- | --- | --- | --- | --- | --- |
| First dose |  |  |  |  |  |  |  |
|  | Crude Model 1^a^ |  |  |  |  |  |  |
|  |  | Thalassemia |  |  |  |  |  |
|  |  | No | - | - | 1.00 | - | - |
|  |  | Yes | -1.681 | 7.018 | 0.186 | 0.054～0.646 | **0.008** |
|  | Adjusted Model 1^b^ |  |  |  |  |  |  |
|  |  | Thalassemia |  |  |  |  |  |
|  |  | No | - | - | 1.00 | - | - |
|  |  | Yes | -1.638 | 6.512 | 0.194 | 0.055～0.684 | **0.011** |
| Second dose |  |  |  |  |  |  |  |
|  | Crude Model 2^a^ |  |  |  |  |  |  |
|  |  | Thalassemia |  |  |  |  |  |
|  |  | No | - | - | 1.00 | - | - |
|  |  | Yes | -1.600 | 4.219 | 0.202 | 0.044～0.929 | **0.040** |
|  | Adjusted Model 2^c^ |  |  |  |  |  |  |
|  |  | Thalassemia |  |  |  |  |  |
|  |  | No | - | - | 1.00 | - | - |
|  |  | Yes | -1.596 | 4.092 | 0.203 | 0.043～0.952 | **0.043** |

The results cannot be shown for the third dose because the sample size of children with adverse reactions after the third dose of hepatitis B vaccination was too small.

^a^No adjustment for confounding factors.

^b^Adjusted for the following factors: Age, Sex, Delayed first vaccination, Premature.

^c^Adjusted for the following factors: Age, Sex, Delayed first vaccination.
