# Supplementary material for: Evaluation of the immunization efficacy and adverse reactions of hepatitis B vaccination in children with thalassemia minor
Source: BMC Public Health. 2024 Sep 27;24:2641. doi: 10.1186/s12889-024-18779-1 (PMC11438186; doi:10.1186/s12889-024-18779-1)
Supplement: Supplementary file 2 — Supplementary Material 2 [file 12889_2024_18779_MOESM2_ESM.doc]

**Questionnaire**

**Basic information on children**

Vaccination File No:________

Child's name:_____ Child's sex:_____ Date of birth:_____

Current child weight: _______kg; height: _______cm

Nation:_______ Parents' valid contact information:_______

1. Was the child infected with HBV at birth? A.Yes B.No
2. Does the child have an immunodeficiency disease? A.Yes B.No
3. Does the child have thalassemia?

A.Yes (Please fill in the type of thalassemia:_______) B.No

**Information on the mother's current pregnancy**

1. Age at current pregnancy _____
2. Was there any preeclampsia in this pregnancy? A.Yes B.No
3. Current mode of delivery. A. Natural birth B. Caesarean section
4. Week of gestation for this delivery: ________
5. Was this delivery premature? A.Yes B.No

**Hepatitis B Vaccination Information for Children**

*(Please refer to the Childhood Immunization Certificate to fill in the following information)*

|  | **vaccination time** | **Vaccination type** | **vaccination dose** | **Vaccination site** |
| --- | --- | --- | --- | --- |
| First dose | **____/____/____** |  | 10ug |  |
| Second dose | **____/____/____** |  | 10ug |  |
| Third dose | **____/____/____** |  | 10ug |  |

**Event of adverse reaction after hepatitis B vaccination**

| **First dose** | | | | |
| --- | --- | --- | --- | --- |
| **Event** |  |  | **Time of symptom onset** | **The duration of symptoms** |
| Injection-site redness and swelling | NO | YES |  |  |
| Injection-site induration | NO | YES |  |  |
| Injection-site pain | NO | YES |  |  |
| Fever | NO | YES |  |  |
| Fatigue | NO | YES |  |  |
| Diarrhea | NO | YES |  |  |
| Others:_________ | | | | |

1. Treatment measures taken after the onset of symptoms.
2. Self-medication B. Seek medical treatment C. Physical cooling
3. No treatment E. Others:_____

| **Second dose** | | | | |
| --- | --- | --- | --- | --- |
| **Event** |  |  | **Time of symptom onset** | **The duration of symptoms** |
| Injection-site redness and swelling | NO | YES |  |  |
| Injection-site induration | NO | YES |  |  |
| Injection-site pain | NO | YES |  |  |
| Fever | NO | YES |  |  |
| Fatigue | NO | YES |  |  |
| Diarrhea | NO | YES |  |  |
| Others:_________ | | | | |

1. Treatment measures taken after the onset of symptoms.
2. Self-medication B. Seek medical treatment C. Physical cooling

D. No treatment E. Others:_____

| **Third dose** | | | | |
| --- | --- | --- | --- | --- |
| **Event** |  |  | **Time of symptom onset** | **The duration of symptoms** |
| Injection-site redness and swelling | NO | YES |  |  |
| Injection-site induration | NO | YES |  |  |
| Injection-site pain | NO | YES |  |  |
| Fever | NO | YES |  |  |
| Fatigue | NO | YES |  |  |
| Diarrhea | NO | YES |  |  |
| Others:_________ | | | | |

1. Treatment measures taken after the onset of symptoms.
2. Self-medication B. Seek medical treatment C. Physical cooling

D. No treatment E. Others:_____
